# Characterizing the effect of GalNAc and phosphorothioate backbone on binding of antisense oligonucleotides to the asialoglycoprotein receptor

**DOI:** 10.1093/nar/gkx060

**Published:** 2017-02-03

**Authors:** Karsten Schmidt, Thazha P. Prakash, Aaron J. Donner, Garth A. Kinberger, Hans J. Gaus, Audrey Low, Michael E. Østergaard, Melanie Bell, Eric E. Swayze, Punit P. Seth

**Affiliations:** Ionis Pharmaceuticals Inc., 2855 Gazelle Court, Carlsbad, CA 92010, USA

## Abstract

Targeted delivery of antisense oligonucleotides (ASO) to hepatocytes via the asialoglycoprotein receptor (ASGR) has improved the potency of ASO drugs ∼30-fold in the clinic (1). In order to fully characterize the effect of GalNAc valency, oligonucleotide length, flexibility and chemical composition on ASGR binding, we tested and validated a fluorescence polarization competition binding assay. The ASGR binding, and *in vitro* and *in vivo* activities of 1, 2 and 3 GalNAc conjugated single stranded and duplexed ASOs were studied. Two and three GalNAc conjugated single stranded ASOs bind the ASGR with the strongest affinity and display optimal *in vitro* and *in vivo* activities. 1 GalNAc conjugated ASOs showed 10-fold reduced ASGR binding affinity relative to three GalNAc ASOs but only 2-fold reduced activity in mice. An unexpected observation was that the ASGR also appears to play a role in the uptake of unconjugated phosphorothioate modified ASOs in the liver as evidenced by the loss of activity of GalNAc conjugated and unconjugated ASOs in ASGR knockout mice. Our results provide insights into how backbone charge and chemical composition assist in the binding and internalization of highly polar anionic single stranded oligonucleotides into cells and tissues.

## INTRODUCTION

Modification of therapeutic agents with ligands that mediate binding to specific surface receptors and facilitate productive uptake is a key strategy for targeted delivery of therapeutics to specific tissues and cells. The surface receptor of high interest for targeted delivery of therapeutic agents to liver is the asialoglycoprotein receptor (ASGR). ASGR is a C-type lectin that is abundantly expressed on the cell surface of hepatocytes in the liver (2). The functional receptor is a multimer comprised of two distinct proteins (ASGR-1 and ASGR-2), and binds terminal galactose (Gal) and *N*-acetyl galactosamine (GalNAc) residues in a calcium-dependent manner (3). The ASGR1 possesses the carbohydrate recognition domain (CRD) for calcium mediated sugar binding (3) and the cytoplasmic signal for binding clathrin adaptor proteins within coated pits (4). Mice lacking the ASGR1 subunit do not bind ligand and do not express ASGR2 on the plasma membrane (5). In contrast, mice lacking the ASGR2 subunit are viable and fertile but express the ASGR1 subunit at reduced levels (6).

The interaction of ASGR with its ligands is well studied and shown to occur in clathrin-coated pits on the basolateral membrane of hepatocytes (7). Upon binding, the ligand–receptor complex is internalized by clathrin-mediated endocytosis (8). Subsequently, acidic pH triggers dissociation of the ligand–receptor interaction in late endosomal compartments (2,9). Recycling endosomes replace ASGR back to the surface of the plasma membrane and make it available for constitutive endocytosis and recycling (2,10). Galactose and GalNAc ligands display maximum affinity to ASGR, and numerous natural and synthetic sugar-based ligands have been described and investigated for their ASGR binding activity (11). For example, a number of biomolecules such as modified lipoprotein particles (12), genes (13) and chemically modified oligonucleotides (14–18) have been studied for targeted delivery via ASGR in animal models.

Antisense oligonucleotides (ASOs)—short, chemically modified oligonucleotides—are attractive candidates for targeted therapeutic delivery via ASGR (17,18). ASOs produce a pharmacological effect by binding to complementary RNA and modulating its function (19). Second-generation ASOs are fully phosphorothioate (PS)-modified (20) chimeric ASOs, and represent the most advanced oligonucleotides in preclinical and clinical development for therapeutic delivery (17). Kynamro™, which is the first systemically delivered second-generation ASO, has been recently approved by the Food and Drug Administration (FDA) for the treatment of homozygous familial hypercholesterolemia (21). There are presently over 35 second-generation ASOs in clinical development, about half of these ASOs target genes primarily expressed by hepatocytes in the liver. Diseases targeted by these ASOs include cardiovascular, diabetes, cancer, and rare and orphan diseases (17,22).

We recently showed that conjugation of tri-antennary GalNAc ligands to ASOs enhances potency 10–60-fold for suppressing gene targets expressed in hepatocytes (17,23). Further structure activity relationship studies revealed that while the tri-antennary GalNAc ligands were optimal, significant changes could be made to the linking scaffolds to reduce structural complexity for ease of manufacturing of GalNAc-conjugated ASO drugs (23). Somewhat surprisingly, our SAR studies also revealed that one and two GalNAc ligands were almost as effective at enhancing ASO potency in the liver (24). Preliminary experiments suggested that chemical features of second-generation ASOs were contributing to enhancing the ASGR binding properties of one GalNAc ASOs (24).

For the studies above, we measured displacement of ^125^I-α1-acid glycoprotein (AGP)—a reported high affinity ligand for the ASGR—to determine the receptor binding properties of GalNAc-conjugated ASOs (17). While this assay was effective, we saw the need for a more efficient binding assay, with a higher throughput, being more cost-effective and avoided the use of radioactive materials. To address these issues, we established a competition binding assay which measures the change in fluorescence polarization upon displacement of a tri-antennary GalNAc Cy5 fluorophore-modified tracer from ASGR containing liver microsomes (25). A further advantage of this assay is the ability to measure species-specific ASGR binding of GalNAc ASO conjugates using commercially available liver microsome preparations.

In this manuscript, we determined the contribution of GalNAc valency, oligonucleotide length, flexibility and chemical composition of the sugar-phosphate backbone, on the overall ASGR binding of GalNAc-conjugated ASOs. We found that while binding of three GalNAc ligands remained largely unaffected by these variables, binding of one and two GalNAc ASOs was influenced by the design of the ASO. An unexpected observation was that both, GalNAc conjugated and unconjugated ASOs showed reduced activity in ASGR knockout mice suggesting that the ASGR is potentially also a pathway for the uptake of *unconjugated* ASOs into hepatocytes. Our observations provide insights into interactions of oligonucleotide conjugates with cell-surface receptors and into potential pathways of cellular uptake for chemically modified ASOs and ASO-conjugates into cells.

## MATERIALS AND METHODS

### Synthesis of 3΄-GalNAc-conjugated ASOs

The GN3 loaded polystyrene solid support was prepared as described previously (16,17). ASOs were synthesized at 40 μmol scale using UnyLinker™ support functionalized by modified nucleoside or GalNAc cluster. For the synthesis of GalNAc-conjugated ASOs, 0.1 M solutions of all phosphoramidites in acetonitrile, and standard oxidizing and capping reagents were used. For each ASO structure, 4-fold excess of amidite was delivered with a 12-min coupling time. The 5΄-end dimethoxytrityl group was left on to facilitate purification. To remove cyanoethyl protecting groups from the phosphorothioate (PS) linkages, all ASOs were treated post-synthetically with 1:1 triethylamine: acetonitrile. Subsequently, ASOs were treated with aqueous NH_4_OH at 55°C for 9–12 h to cleave from support, remove protecting groups, and hydrolyze the UnyLinker™ moiety. ASOs were purified by ion-exchange chromatography using a gradient of NaBr across a column packed with Source 30Q resin. Pure fractions were desalted using high performance liquid chromatography (HPLC) on a reverse phase column. Purity and mass of ASOs were determined using ion-pair liquid chromatography–mass spectrometry (LC–MS) analysis (Supplementary Table S1).

### Synthesis of 5΄-GalNAc-conjugated ASOs in solution

Synthesis of 5΄-GN1, GN2 and GN3-conjugated ASOs were achieved in 60–78% isolated yield according to the reported procedure (18,23,24). 5΄-hexylamino ASOs were dissolved in 0.1 M sodium tetraborate buffer, pH 8.5 (2 mM) and a solution of GalNAc PFP ester (3 mol equivalent) dissolved in DMSO (40 mM) was added. The reaction mixture was stirred at room temperature for 3 h. To this aqueous ammonia (28–30 wt%) was added (5× reaction volume) and stirred at room temperature for 4 h. Reaction mixture concentrated under reduced pressure and residue dissolved in water and purified by HPLC on a strong anion exchange column (GE Healthcare Bioscience, Source 30Q, 30 μm, 2.54 × 8 cm, A = 100 mM ammonium acetate in 30% aqueous CH_3_CN, B = 1.5 M NaBr in A, 0–60% of B in 60 min, flow 14 ml/min). The residue was desalted by HPLC on reversed phase column to yield the 5΄-GN1, GN2 and GN3 conjugated ASOs. The ASOs were characterized by ion-pair-HPLC–MS analysis (Supplementary Table S1).

### Synthesis of linear 1, 2 and 3 GalNAc-conjugated ASOs

ASOs containing 1–3 hydroxy-proline (HP) GN modifications were synthesized according to the procedure reported (23). In brief ASOs were synthesized at 40 μmol scale using UnyLinker™ solid support. For the synthesis of 3΄-HP-GalNAc-conjugated ASOs 0.1 M solution of GalNAc phosphoramidite (18,23) in 40% dichloromethane in acetonitrile was used. First phosporamidites coupled on a UnyLinker™ solid support and subsequently required nucleoside phosphoramidites were coupled to synthesize the 3΄-HP-GalNAc-conjugated ASOs. For 5΄-HP-GalNAc conjugation required ASO was first assembled on a solid support and HP-GalNAc phosphoramidite was coupled at the 5΄-end to complete the synthesis (23). A solution of all other phosphoramidites in acetonitrile (0.1 M), and standard oxidizing and capping reagents were used (23). Post-synthetically, all oligonucleotides were treated with 1:1 triethylamine:acetonitrile to remove cyanoethyl protecting groups from the phosphorothioate linkages. Subsequently, solid support bearing ASOs were treated with aqueous NH_4_OH (28–30 wt%) at 55°C for 4 h and then cooled and added 10% (v/v) of 40% methylamine in water. Heating at 55°C was continued for additional 12–14 h to cleave GalNAc-conjugated ASOs from support, remove protecting groups, and hydrolyze the UnyLinker™ moiety. Oligonucleotides were purified by ion-exchange chromatography using a gradient of NaBr across a column packed with Source 30Q resin as described before. Pure fractions were desalted using HPLC on a reversed phase column. Purity and mass of oligonucleotides were determined using ion-pair LC–MS analysis (Supplementary Table S1).

### Microsome preparation

Microsome fractions containing ASGR were enriched from fresh or frozen Balb/C mouse livers as previously described (25,26). Briefly, livers were thawed on ice and homogenized without frothing in 3 volumes of cold homogenization buffer (0.1 M Tris–HCl, pH 7.5, with 10 mM EDTA, 150 mM KCl and Roche complete mini protease inhibitor cocktail). The homogenate was centrifuged at 12 500 g for 15 min. The resulting supernatant was ultra-centrifuged at 105 000 g for 70 min. The pellet was resuspended and homogenized in 2 volumes of cold wash buffer (0.1 M Tris–HCl, pH 7.5, with 10 mM EDTA and Roche complete mini protease inhibitor cocktail) and then ultra-centrifuged for 45 min at 105 000 g. The pellet was resuspended and homogenized in 1.5 volumes of cold microsome buffer (0.05 M Tris–HCl, pH 7.5, with 10 mM EDTA, 20% glycerol, and Roche complete mini protease inhibitor cocktail). Protein concentration was determined by Quant-iT protein assay kit (Thermo Fisher). ASGR was quantified using western blot and and mass spectrometry using AQUA peptides (data not shown). Samples were aliquoted and stored at –80°C. Microsome preparation was scaled up so that all assays could be performed with the same lot of ASGR membranes. Commercially available microsome fractions from human and monkey livers were obtained from Invitrogen Corp. (Carlsbad, CA, USA) and BioreclamationIVT (Hicksville, NY, USA).

### Synthesis of GN3-SulfoCy5 Tracer (S3)

Synthesis of Tracer was accomplished as described in supplementary scheme S1. GalNAc cluster Pfp ester S1 was synthesized using reported procedure (23). To a solution of GalNAc cluster Pfp ester S1 (97 mg, 51 μmol) in dichloromethane (170 μl) a solution of *N*-Boc-1,3-diaminopropane (52.8 mg, 303 μmol) in dichloromethane (50 μl), and triethylamine (7 μl, 95 μmol) were added. After stirring for 3 h the reaction mixture was diluted with dichloromethane (50 ml) and washed with 1 M aqueous NaHSO_4_ solution (3 × 50 ml), aqueous saturated sodium bicarbonate (2 × 25 ml) and brine (50 ml). Organic phase separated, dried (Na_2_SO_4_) and concentrated. The residue obtained was dissolved in dichlormethane (3 ml). To this trifluoroacetic acid (1 ml) was added and the reaction mixture was stirred at room temperature for 1 h. Reaction mixture was diluted with toluene (10 ml) and evaporated under reduced pressure. The residue thus obtained was dissolved in acetonitrile (2 ml) and co-evaporated with toluene (2 ml) under reduced pressure to yield GalNAc cluster amine S2 (Scheme S1). LRMS (ES, positive) *m/z* calculated for C_81_H_134_N_9_O_35_ [M+H]^+^: 1794.0, found 1793.8. GalNAc cluster amine S2 (92.7 mg, 48.6 μmol) was dissolved in DMF (250 μl) and Cy5-NHS-Ester (55.3 mg, 72.9 μmol (Scheme S1) was added. To this triethylamine (28 μl, 200 μmol) was added and allowed to shake for 18 h. The reaction mixture was concentrated under reduced pressure. To the residue obtained aqueous ammonia (1.5 ml, 28–30 wt%) was added and vortexed to get a solution and resulting solution was allowed to shake at room temperature for 2 h. The reaction mixture was diluted with water (20 ml) and purified by HPLC on a reverse phase column to yield GN3-SulfoCy5 Tracer S3 (5.28 mg, 6.5%, Scheme S1). ^1^H NMR (300 MHz, D_2_O) δ 1.17 (br s, 12H), 1.26–1.64 (m, 29H), 1.66-1.78 (m, 4H), 1.82 (s, 2H), 1.88–1.96 (m, 10H), 2.05–2.18 (m, 9H), 2.33 (br t, *J* = 5.63 Hz, 6H), 2.95–3.10 (m, 10H), 3.39–3.47 (m, 3H), 3.50–3.84 (m, 35H), 4.02 (br s, 2H), 4.31 (d, *J* = 8.32 Hz, 3H), 6.22 (br d, *J* = 13.19 Hz, 2H), 6.49–6.57 (m, 1H) 7.27 (d, *J* = 8.32 Hz, 2H), 7.72–7.80 (m, 4H), 8.01 (s, 2H); LRMS (ES, positive) *m/z* calculated for C_95_H_153_N_11_O_33_S_22_ [M+H]^+^: 2039.4, found 2039.5.

### Fluorescence polarization (FP) binding assay

We adapted assay conditions reported by Kornilova with a few changes outlined (25). Instead of a GN3-Cy5 tracer we utilized a GN3-sulfoCy5 tracer S3 as described above due to reduced non-specific binding and increased dynamic range. Reagents were combined to a total of 100 μl per well of a 96-well black-walled Nunc OptiPlate (PerkinElmer). 40 μg of microsomes were diluted to 43.5 μl in 50 mM Tris buffer at pH 7. Microsomes were then combined with 24 μl of 5× reaction buffer consisting of 125 mM Tris–HCl (pH 7.8), 12.5 mM CaCl_2_, 5 mg/ml bovine serum albumin (BSA), and 0.1% Triton X-100. 10 μl of GalNAc ASO of various concentrations was added to the mixture and pre-incubated for 15 min. Then, 12.5 μl GN3-sulfoCy5 tracer S3 was added to the mixture for a final concentration of 1.25 nM (25). The assay was incubated for additional 60 min at room temperature with agitation. Readings were taken using the Tecan Infinite M 1000 Pro instrument (λ_ex_ = 635 nm, λ_em_ = 675 nm). Using polarized excitation and emission filters, the instrument measures fluorescence perpendicular to the excitation plane (the ‘P-channel’) and fluorescence that is parallel to the excitation plane (the ‘S-channel’), and then it calculates FP in millipolarization units (mP) as follows:

mP = [(S – P _*_ G) / (S + P _*_ G)] _*_ 1000. The ‘G-factor’ is measured by the instrument as a correction for any bias toward the P channel. Specific binding was calculated by subtraction of the mP value of the highest competitor concentration utilized after addition of galactose to a final concentration of 1 mM.

### Primary hepatocyte cell culture

Freshly isolated mouse hepatocytes were placed in wells with growth medium containing 10% FBS, antibiotic-antimycotic, HEPES, glutamine and varying amounts of ASO or PBS control. Cells were maintained at 37°C and 5% CO_2_ for 16 h, and then washed with PBS and lysed. RNA was extracted using Qiagen RNeasy kit and SRB1 mRNA levels determined by Taqman *q*-rtPCR using the primers: 5΄-TGACAACGACACCGTGTCCT-3΄ (forward primer), 5΄-ATGCGACTTGTCAGGCTGG-3΄ (reverse primer) and 5΄-CGTGGAGAACCGCAGCCTCCATT-3΄ (probe). RNA was normalized to total RNA using RiboGreen, and all experiments were performed in triplicate.

### Animal treatment

Animal experiments were conducted in accordance with the American Association for the Accreditation of Laboratory Animal Care guidelines and were approved by the Animal Welfare Committee (Cold Spring Harbor Laboratory's Institutional Animal Care and Use Committee guidelines). The animals were housed in microisolator cages on a constant 12-h light–dark cycle with controlled temperature and humidity, and were given access to food and water *ad libitum*. Tissues were collected, weighed, flash frozen on liquid nitrogen, and stored at –60°C.

#### Knockout mice

ASGR1 –/– (JAX#009105) and their respective controls, C57BL/6NJ (JAX#005304), were obtain from The Jackson Laboratory. 5–6 mice/group were dosed by subcutaneous injection of saline or ASO 38 at 0.3, 1, 3 and 10 mg/kg or ASO 39 at 3, 8 and 24 mg/kg once and sacrificed 72 h after dosing. A 6 mm biopsy punch was used to remove an ∼50–100 mg piece of liver tissue, the tissue was placed in 1.5 ml of 1% beta-mercaptoethanol in buffer RLT (Qiagen) and homogenized for 10 s with a TH-01 tissue homogenizer (Omni International) at 35 000 rpm. The homogenized liver tissue was placed on dry ice to freeze the homogenate and stored at –80°C until the RNA purification was performed. GCGR mRNA expression was analyzed by qRT-PCR using the OneStep RT-PCR system (ThermoFisher/Life Technologies, Grand Island, NY, USA). The sequences of primers and probe used for mouse glucagon receptor (GCGR) are 5΄-ATTTCCTGCCCCTGGTACCT-3΄ for the forward primer, 5΄-CGGGCCCACACCTCTTG-3΄ for the reverse primer, and 5΄-CCACAAAGTGCAGCACCGCCTAGTGT-3΄ for the probe.

#### Scavenger receptor B1 mouse protocol

Six- to eight-week-old C57BL/6 mice (Charles River Laboratories) were treated according to the indicated treatment schedules. A 6 mm biopsy punch was used to remove an ∼50–100 mg piece of liver tissue, the tissue was placed in 2 ml of 8% beta-merccaptoethanol in UltraPure™ Guanidine Isothiocyanate Solution (Thermo Fisher) and homogenized for 10 s with a TH-01 tissue homogenizer (Omni International) at 35 000 rpm. The homogenized liver tissue was placed on dry ice to freeze the homogenate and stored at –80°C until the RNA purification was performed. The sequences of primers and probe used for mouse scavenger receptor B1 (SRB1) are 5΄-TGACAACGACACCGTGTCCT-3΄ for the forward primer, 5΄-ATGCGACTTGTCAGGCTGG-3΄ for the reverse primer, and 5΄-CGTGGAGAACCGCAGCCTCCATT-3΄ for the probe.

### Quantitative RT-PCR

Reduction of target expression was determined by reverse transcription-quantitative polymerase chain reaction (RT-qPCR) using the StepOnePlus System (Thermo Fisher). Briefly, RNA was extracted from ∼50–100 mg of liver tissue from each mouse using a 96-well format RNeasy Kit (Qiagen) or the PureLink^®^ Pro 96 total RNA Purification Kit (Thermo Fisher) with on column DNase I (Thermo Fisher) treatment and target mRNA was measured by RT-qPCR using the Express StepOne SuperMix Kit (Thermo Fisher). Part of the initial total RNA eluates were diluted to a concentration of approximately 10 ng/μl and the actual concentration was determined using the Quant-IT Ribogreen RNA assay (Thermo Fisher). Approximately 50 ng of total RNA was combined with 10 μl Express PCR Supermix, target specific forward and reverse primers and hydrolysis probe (at 375, 375 and 125 nM, respectively; Integrated DNA Technologies), 0.4 μl ROX Reference Dye and 2 μl of Express Superscript Mix in a total reaction volume of 20 μl in a 96-well PCR plate. Samples were run on StepOnePlus thermocyclers with the following cycling protocol: 15 min at 50°C, 2 min at 95°C and 40 cycles of 15 s at 95°C and 1 min at 60°C. C_q_ values were derived using the StepOne software v2.3 and relative levels of mRNA expression for each sample was determined by comparing the sample C_q_ to the C_q_ values of a dilution series (serial 2-fold or 4-fold dilutions) of concentrated RNA, from a pool of the initial RNA eluates. Relative target expression was then normalized to total input RNA levels based on the Ribogreen assay values. The normalized sample values were then divided by the average value of all samples from the appropriate control group to generate the reported relative expression of the target mRNA. Data are mean values ± standard deviations.

### Ki, IC_50_, ED_50_ and statistical significance determination

Inhibition constants (*K*_i_ values) were determined with GraphPad Prism 5 software using non-linear regression for curve fit assuming one binding site with one concentration of ligand and multiple concentrations of competitor. Concentration and dissociation constants (*K*_D_) of tracer were constrained to 1.25 and 9 nM, respectively. Effective doses to cause 50% inhibition (ED_50_) values were determined by plotting log dose versus mRNA relative to untreated controls using a four-parameter fit with variable slope and constraining bottom = 0 and top = 1. Statistical significance of *in vivo* knockdown experiment in ASGR knockout mice were calculated using a two-way ANOVA analysis.

## RESULTS

### GN3-SulfoCy5 tracer (S3) is the optimal tracer for fluorescence polarization assay

**Figure 1.**
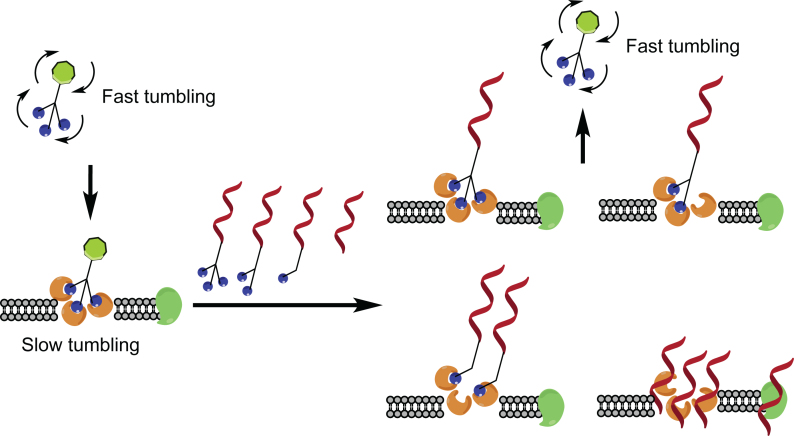
Physical principle of fluorescence polarization (FP) competition assay to measure the binding of one, two and three GalNAc-conjugated ASOs to ASGR. FP can be used to monitor binding of GN3-SulfoCy5 Tracer (S3) to ASGR in solution. GN3-SulfoCy5 Tracer (S3) excited with plane-polarized light emits generally depolarized light because the tracer tumbles fast during the time between excitation and emission resulting in low polarization. Upon binding to ASGR, the GN3-SulfoCy5 Tracer (S3) rotates slower and the emitted light remains polarized leading to higher polarization. Competition with one, two or three GalNAc-conjugated ASOs reverses the effect and therefore provides a direct readout of binding to ASGR.

The physical principle of the FP assay is depicted in Figure 1. Displacement of a membrane-bound GN3-SulfoCy5 tracer S3 with a GalNAc ASO leads to a change in fluorescence polarization as a consequence of enhanced Brownian motion of the displaced tracer in solution (27). Criteria for a workable tracer are minimal non-specific binding and a sufficient large dynamic range. To utilize the best suited tracer molecule, we tested a series of potential candidates, including mono- and trivalent GalNAc cluster (GN3-Cy5, GN3-SulfoCy5, GN1-Cy5, GN1-SulfoCy5) and a 3΄-SulfoCy5-labeled ASO attached to a 5΄-mono-valent GalNAc-cluster (GN1-ASO-SulfoCy5) (data not shown). GN3-SulfoCy5 tracer was found to have minimal nonspecific binding to mouse liver microsomes and an acceptable dynamic range (Supplementary Figure S1) (28).

### Design of GalNAc ASO conjugates to determine importance of GalNAc valency and spatial arrangement for ASGR binding

**Figure 2.**
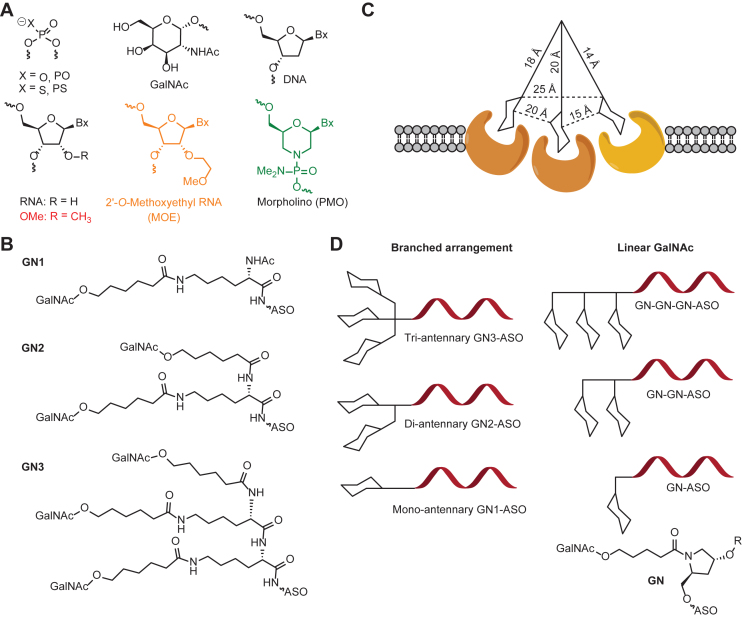
(**A**) Structures of nucleoside modifications used in ASOs. (**B**) Structures of one, two and three GalNAc conjugates. (**C**) Accepted model of ASGR binding for a tri-antennary scaffold with precise distances between the GalNAc sugars (11). (**D**) Schematic of branched versus linear arrangement of GalNAc–ASO conjugates used in the present study.

For the present study we synthesized ASOs targeting the Scavenger Receptor Class B 1 (SRB-1) mRNA using chemistries described in Figure 2A. The 5–10–5 MOE gapmer ASOs were designed with variable amounts of phosphorothioate (PS) and phosphodiester (PO) linkages along with different arrangements of one, two and three GalNAc sugars (Figure 2B and Supplementary Table S1).

**Figure 3.**
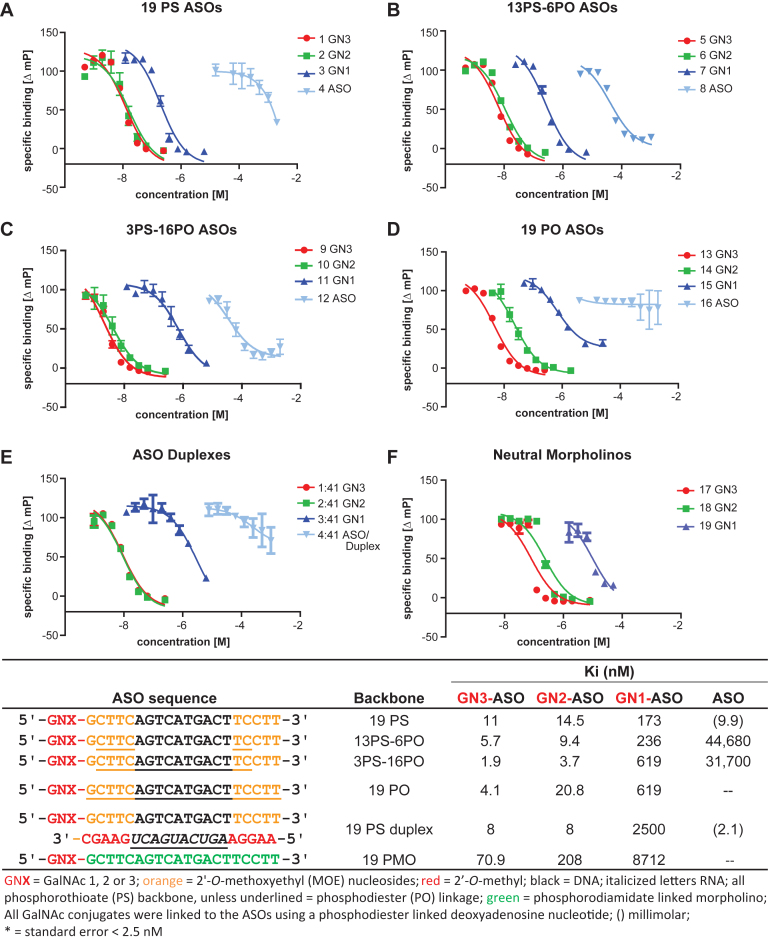
Effect of oligonucleotide backbone composition on ASGR binding of one, two and three GalNAc ASOs: (**A**) 19 PS-0 PO, (**B**) 13 PS-6 PO, (**C**) 3 PS-16 PO and (**D**) 19 PO linkages. Influence of (**E**) duplexation and (**F**) charge on ASGR binding of one, two and three GalNAc ASOs.

**Figure 4.**
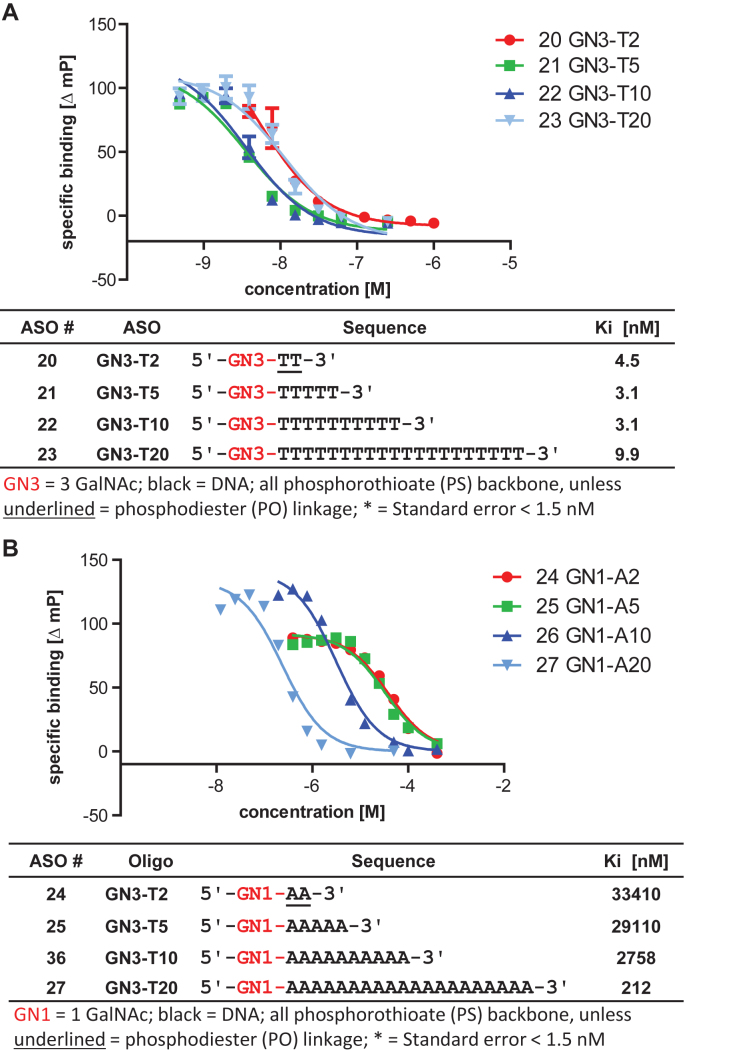
Effect of length on the ASGR binding of (**A**) three GalNAc and (**B**) one GalNAc conjugated 2-mer, 5-mer, 10-mer and 20-mer oligonucleotides.

A widely accepted model for ASGR binding proposes a tri-antennary scaffold with precise distances between the sugar moieties and the linker scaffold as the optimal ligand (11,29) (Figure 2C). To determine if this model is applicable for GalNAc-conjugated ASO drugs, we examined the importance of the tri-antennary scaffold and linear spatial orientation of GalNAc sugars on ASGR binding in the FP assay (Figure 2D). To accomplish this, the 1 GalNAc ligand was attached to the 5΄- or the 3΄-end of the ASO via sequential phosphoramidite coupling as described previously (23,30). This allows the GalNAc ligands to be displayed in a linear as opposed to a tri-antennary arrangement presented by the two and three GalNAc ligands used for the studies described in Figures 3 and 4 (23). In addition, we also evaluated the effect of attaching GalNAc sugars on either ends of single stranded and duplex ASOs. This allows for better control over the orientation and distance between the GalNAc sugars, as oligonucleotide duplexes have well-defined helical structures.

### ASGR binding of GalNAc-ASOs is affected by single-strandedness, chemical composition and charge of the oligonucleotide backbone

First, we examined the effect of ASO single-strandedness, backbone charge and chemical composition on ASGR binding in the FP competition binding assay. To evaluate the effect of backbone composition, ASOs containing full phosphorothioate (19 PS, **1–4**, Figure 3A) a combination of phosphorothioate (PS) and phosphodiester (PO) linkages (13 PS-6 PO, **5–8**, Figure 3B and 3 PS-16 PO, **9–12**, Figure 3C) or phosphodiester (19 PO, **13–16**, Figure 3D) backbones, each conjugated to one, two or three GalNAc sugars were tested (Supplementary Table S1). The PS linkage is a commonly used modification to enhance stability from nuclease-mediated degradation and protein binding properties of oligonucleotide drugs. The mixed PS/PO backbone ASO designs have recently been employed to mitigate the unintended protein binding of PS ASOs and enhance potency in the liver when conjugated to tri-antennary GalNAc ligands (23).

For full PS ASOs, two and three GalNAc ASOs showed 10-fold improvement in ASGR binding affinity relative to the one GalNAc ASO (Figure 3A). Surprisingly, we observed competition of the unconjugated parent ASO with the GN3-sulfoCy5 tracer, albeit at very high concentrations. One possible explanation is that the unconjugated ASO does not interact with the receptor within the carbohydrate binding pocket where GalNAc binds, but instead interacts with the receptor in a non-specific manner causing displacement of the GalNAc tracer S3 from the shallow binding pocket in ASGR1 (Figure 1).

Replacing PS with PO/PS backbone had a small effect on binding for the two and three GalNAc ASOs, but a substantial loss of binding was observed for the weaker binding one GalNAc ASOs compared to the full-PS counterparts (Figure 3B and C). In the absence of any PS linkages, one and two GalNAc ASOs showed decreased binding compared with the three GalNAc ASO, whereas the binding of the unconjugated ASO was completely abolished (Figure 3D).

To evaluate the effect of backbone flexibility on ASGR binding, we tested the 19 PS ASO conjugated to one, two and three GalNAc sugars and duplexed with a phosphodiester RNA complementary strand (31). Single stranded ASOs are conformationally more flexible and may have differential abilities to interact with a surface bound protein as compared to oligonucleotide duplexes which have more defined helical structures. Two and three GalNAc ASO–RNA duplexes showed tight bind to microsomes. In contrast, the one GalNAc ASO–RNA duplex showed a 300-fold weaker binding (Figure 3E). However, the binding of one GalNAc ASO–RNA duplex was still 1000-fold stronger than the binding of the unconjugated ASO–RNA duplex.

To determine the influence of backbone charge on ASGR binding, we evaluated morpholino ASOs conjugated to one, two and three GalNAc sugars (Figure 3F). Morpholinos (PMOs) are neutral oligonucleotide analogs where the furanose sugar–phosphate backbone has been replaced with a phosphorodiamidate linked morpholino sugar (32). Loss of charge had an impact on the binding of one, two or three GalNAc Morpholinos (Figure 3F) relative to the designs examined above where the oligonucleotide backbone was negatively charged. Compared with one, two or three GalNAc ASOs with full PS backbones, the binding was reduced by 50-fold, 15-fold and 6-fold, respectively.

### Binding of 1 GalNAc ASO is affected by ASO length

We next determined the role of ASO length on ASGR binding in the competition binding assay. Because the most pronounced differences were observed between one GalNAc and three GalNAc ASOs, we chose to further study these two designs. Increasing length from 2 to 20 nucleotides did not or only marginally affected the binding of three GalNAc ASOs (Figure 4A). In contrast, increasing the ASO length from a 2-mer to a 20-mer had a very significant effect for enhancing the binding of one GalNAc ASOs to microsomes suggesting that oligonucleotide length plays a role in ASGR binding (Figure 4B).

### ASGR binding of double stranded ASOs is affected by the spatial arrangement and valency of GalNAc sugars

To understand the contribution of GalNAc valency and spatial arrangement on ASGR binding and biological activity, we evaluated the effect of attaching one, two and three GalNAc sugars in a linear arrangement, to the 3΄- and/or 5΄-end of single stranded and duplexed ASOs (Table 1). Attaching even a single GalNAc sugar to the 3΄- or the 5΄-end (ASOs **29** and **30**) of the single stranded SRB1 ASO **4**, gave a several thousand-fold enhancement in binding affinity relative to the unconjugated ASO or the monovalent GalNAc sugar itself (*K*_i_ ∼ 860 μM) (11). The improved ASGR affinity also resulted in enhanced activity in primary hepatocytes and in mice for the 1 GalNAc single stranded ASOs **29** and **30** relative to the parent ASO **4**.

**Table 1.** Summary of ASGR binding, *in vitro* and *in vivo* activities of GalNAc-conjugated ASOs to determine the importance of spatial arrangement and GalNAc valency for single and double stranded ASOs

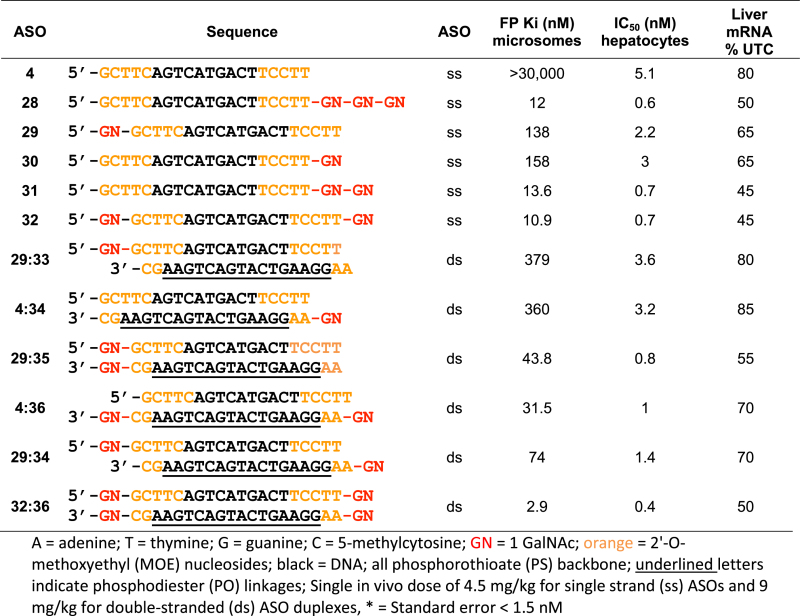

Attaching two GalNAc sugars to the 3΄-end (ASO **31**) or one sugar to the 3΄- and the 5΄-end (ASO **32**) of the ASO resulted in further enhancement in ASGR binding affinity relative to the one GalNAc designs above (ASOs **29–30**). Interestingly, ASOs **31** and **32** showed similar binding affinity and activity as ASO **28** in which the three GalNAc sugars were displayed in a linear arrangement. The improved ASGR binding and activity of ASO **32** with a GalNAc sugar on either end of the oligonucleotide is especially interesting as this arrangement does not conform to the previous model for ASGR binding which proposed the importance of precise orientations and distances between the GalNAc sugars for optimal binding (Figure 2C) (29).

We next evaluated the effect of attaching 1 GalNAc sugar to an ASO duplex (**29:33** and **4:34**) which is functionally active in cells as the complementary phosphodiester strand is cleaved within endosomes to liberate the parent ASO (31). Both these designs showed weaker ASGR binding and activity as compared to the one GalNAc single stranded ASOs, once again suggesting that the backbone flexibility imparted by the single stranded design enhances interactions with the surface receptor.

We also evaluated the effect of introducing two GalNAc sugars in different configurations on the ASO duplexes (**29:35, 4:36, 29:34**). Once more, we observed that GalNAc valency had a greater impact on ASGR binding and activity as compared to the spatial orientation of the GalNAc sugars. Interestingly, the ASO duplex with a GalNAc sugar attached to either end of each strand (**32:36**, four GalNAc sugars), showed the strongest ASGR binding and activity comparable to the single stranded three GalNAc ASO **28** (Table 1).

### Structural models to illustrate interactions of single and double stranded ASOs with the ASGR

**Figure 5.**
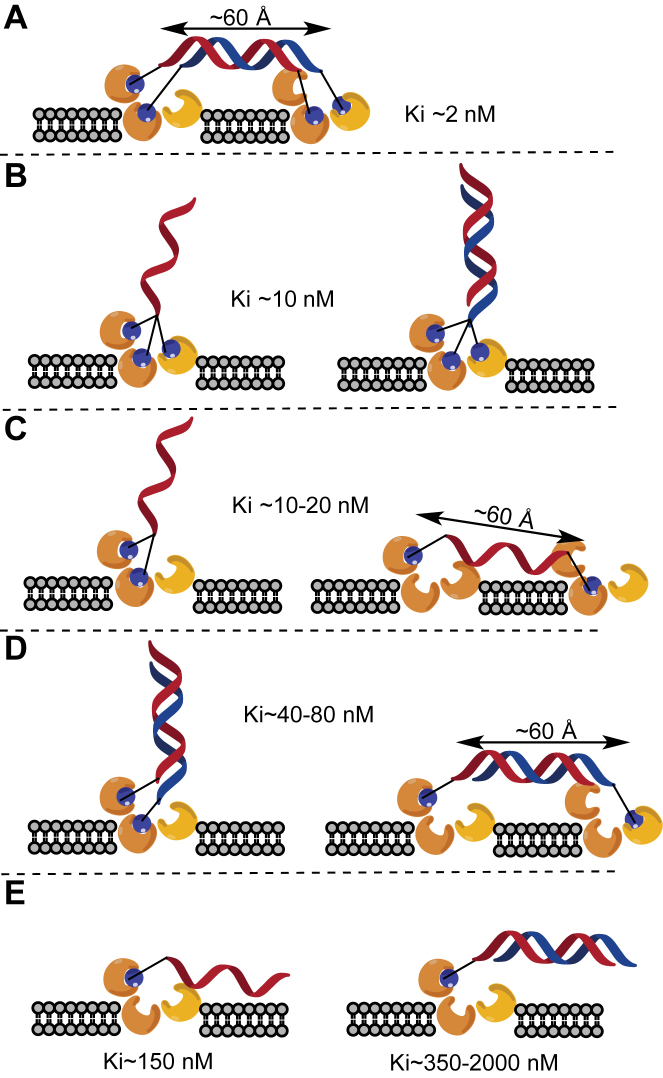
Proposed binding models for GalNAc conjugated single and double stranded ASOs. (**A**) ASO duplex with 1 GalNAc at 3΄ and 5΄ end of each strand; (**B**) single stranded and duplexed ASOs conjugated to three GalNAc; (**C**) single stranded ASO with two GalNAc at one end or one GalNAc at each end; (**D**) duplexed ASO with one GalNAc at opposite ends or one GalNAc at each end and (**E**) one GalNAc conjugated to a single stranded or a duplexed ASO. The length of a 20-mer single or double stranded oligonucleotide was estimated to be 60 Å.

A potential explanation for the SAR data above is illustrated in Figure 5. The generally accepted stoichiometry for ASGR1 and ASGR2 is 2:1 (33). However, others have reported stoichiometries ranging from 2:1 to 5:1 for ASGR1 and ASGR2 based on immunoprecipitation assays (33). Thus, our binding data can be rationalized by models where the relatively long oligonucleotide spacer helps the GalNAc sugars bind multiple receptors on the cell surface or that the functional ASGR is a loosely held aggregate of 2–5 ASGR1 units. The ASO duplex with four GalNAc sugars attached to both ends of each strand, showed the strongest ASGR interaction (Figure 5A). Binding of three GalNAc ASOs was largely unaffected by duplex formation (Figure 5B). Binding of single stranded ASOs with a two GalNAc on one end or one GalNAc on each end was comparable to the 3-GalNAc ASO (Figure 5C). However, binding of two GalNAc ASOs was reduced by duplexation but unaffected by spatial arrangement of the GalNAc sugars (Figure 5D). Lastly, ASGR binding of one GalNAc ASOs was reduced by duplexation suggesting that the single stranded nature enables more robust interactions with the surface receptor (Figure 5E).

### GalNAc conjugated and unconjugated ASOs show reduced activity in ASGR knockout mice

To determine if ASGR mediated potentiation of GalNAc-conjugated ASOs could be measured in cell culture, freshly plated mouse hepatocytes were treated with a three GalNAc-conjugated ASO targeting apoCIII mRNA without any transfection agent to help deliver the oligonucleotide across the plasma membrane. Since fresh hepatocytes undergo rapid de-differentiation in culture, we treated cells with GalNAc conjugated and unconjugated ASOs at 4, 8 and 24 h after plating and also monitored expression of ASGR1 and ASGR2 at these time-points (Supplementary Figure S2). For each time point, cells were treated with ASOs for 16 h prior to analysis of mRNA reduction by qRT-PCR. No loss in activity of the three GalNAc ASO was observed if cells were treated up to 8 h after plating but significant loss in potentiation of antisense activity was observed at the 24 h time point which coincided with loss of ASGR1 and ASGR2 mRNA in these cells (Supplementary Figure S2). Interestingly, loss of ASGR expression also coincided with loss of activity of the unconjugated ASO suggesting that the ASGR could be involved in the functional uptake of the GalNAc-conjugated and unconjugated ASOs into hepatocytes (Supplementary Figure S2).

**Figure 6.**
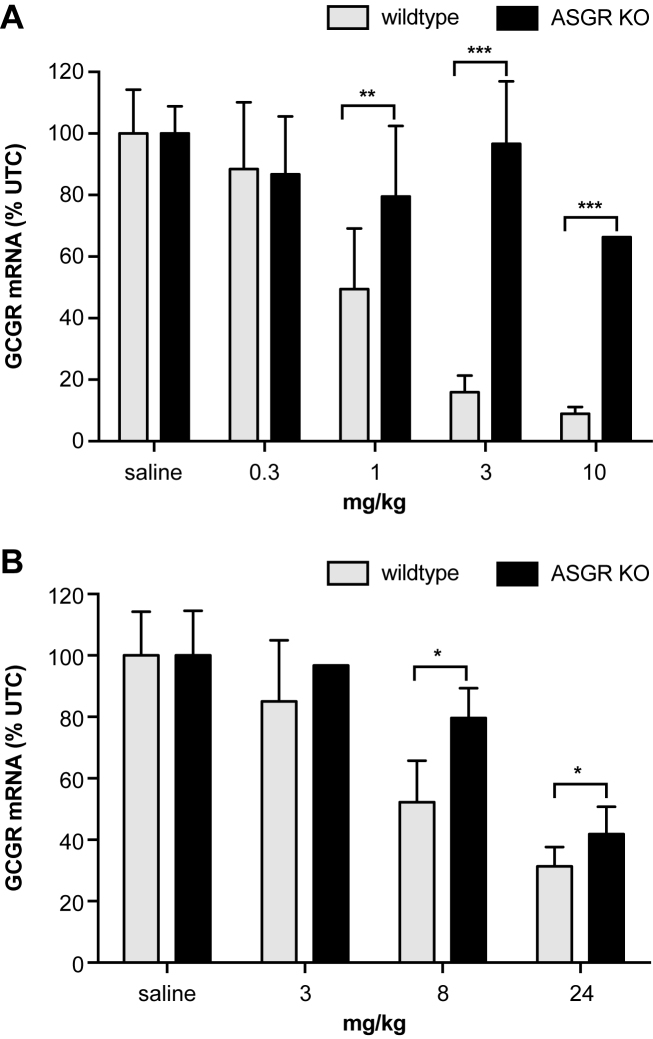
Potency of three GalNAc-conjugated and unconjugated ASOs targeting GCGR mRNA in ASGR knockout and wild type mice. ASGR1 null and wild-type C57BL/6NJ mice were injected subcutaneously with (**A**) three GalNAc ASO **38** at 0.3, 1, 3 and 10 mg/kg and (**B**) with unconjugated ASO **39** at 3, 8 and 24 mg/kg. Mice were sacrificed 72 h after injection, livers were homogenized and reduction of GCGR mRNA in the liver was measured by qRT-PCR. Both, the GalNAc conjugated and unconjugated parent ASOs showed statistically significant reduction in antisense activity in the ASGR knockout mice (*P*-values: * <0.07, ** = 0.003, *** <0.0001).

To confirm the above results in animals, we determined the activity of a three GalNAc ASO and its unconjugated parent ASO, targeting glucagon receptor (GCGR) mRNA in ASGR knockout mice (Figure 6). GCGR was chosen as the target as it is primarily expressed in hepatocytes in the liver. This avoids potential complications arising from targeting genes which are also expressed in non-parenchymal cells of the liver as those would not be targeted by the three GalNAc ASO. For the three GalNAc ASO, there was a dose-dependent knockdown of target mRNA in normal mice, whereas the effect was largely abolished in ASGR-knockout mice at the doses evaluated (Figure 6A). Importantly, confirming our *in vitro* results, dose-dependent knockdown of GCGR mRNA by the unconjugated parent ASO was also reduced significantly in the ASGR-knockout mice (Figure 6B). The ASGR knockout mice were also injected with ASO 1 and GN3-ASO 4 targeting SRB1 mRNA as controls to show that PS ASOs do not reduce GCGR mRNA in a non-specific manner in these mice (Supplementary Figure S3).

## DISCUSSION

### Fluorescence polarization assay for measuring the ASGR binding properties of GalNAc-conjugated ASOs

ASGR is primarily expressed on hepatocytes and therefore is well-suited for receptor-mediated delivery of drugs (2). Target delivery of oligonucleotide-based therapeutics to liver via ASGR represents an important, advanced treatment platform, and specific modifications of oligonucleotides carry a great potential for improving potency for reducing gene targets expressed in hepatocytes. Employing an optimized, high-throughput assay to measure the binding of a ligand to ASGR is crucial, as it allows effective testing and developing of novel and improved therapeutics. We tested and validated a recently developed fluorescence polarization assay (25), and used it for the accurate screening of ASOs with different chemical compositions and configurations of GalNAc sugars. This optimized and validated assay enables quick, efficient, high-throughput screening of ASOs for their ASGR binding properties.

### Structural properties of ASOs influences ASGR binding and antisense activity

In the model for binding to ASGR mediated by GalNAc sugars, single stranded or even duplexed ASOs, conjugated to 3 GalNAc sugars bind the ASGR with the highest affinity, and can undergo ASGR-mediated endocytosis (Figure 5). ASOs conjugated to two or three GalNAc sugars indeed showed the strongest binding and activity *in vitro* and *in vivo*, characterized by ∼10-fold increase in activity compared with unconjugated ASOs. ASGR binding of ASOs conjugated to two or three GalNAc sugars was not affected by PS content, length, charge, or spatial arrangement of GalNAc sugars. In contrast, single strandedness, PS content, length and charge affected the ASGR binding and *in vitro* and *in vivo* activity of one GalNAc ASOs.

It is important to note, that although ASOs conjugated to one GalNAc showed 10-fold reduced binding affinity compared to ASOs conjugated to two or three GalNAc sugars, their *in vivo* activity was reduced by only 2-fold. These results suggest that the ASGR binding affinity of one GalNAc ASOs in the competition binding assay is not directly proportional to their *in vivo* activity which may be a reflection of the higher stoichiometry of one GalNAc ASO ligands required to displace the three GalNAc tracer from ASGR membranes (Figure 1). Furthermore, enhancing the ASGR binding affinity of single stranded one GalNAc ASOs by using modified GalNAc sugars could represent an alternate route for developing GalNAc ASO drugs with simplified chemical structures (34).

### Consequence of ASO participation on ASGR binding

An unexpected outcome from our work was the realization that the ASGR is potentially involved in the uptake of unconjugated PS ASOs into hepatocytes. The importance of the PS modification for enhancing the protein binding properties of oligonucleotides has been long recognized (35,36). We recently described the identification of Stabilin-2—a class H scavenger receptor—as a specific pathway for the uptake of PS ASOs into the sinusoidal endothelial cells in the liver and the spleen (37). Results described in this manuscript suggest that PS ASOs can also interact with other cell surface receptors such as the ASGR and these interactions can have functional consequences on ASO activity in the liver.

Our binding data suggests that the unconjugated ASO does not interact with the receptor within the carbohydrate binding pocket given the high concentrations of unconjugated ASO required to displace the ASGR membrane bound GN3-SulfoCy5 tracer. It is more likely that the ASO interacts with basic amino acids on the exposed surface of the receptor and this interferes with binding of GalNAc within the shallow carbohydrate binding pocket in ASGR1 (Supplementary Figure S4). This hypothesis is supported by the observations that oligonucleotide length, flexibility and backbone charge influence the ASGR binding of GalNAc ASOs.

The X-ray crystal structure of the ASGR1 ectodomain shows that the carbohydrate binding pocket is shallow and solvent exposed (3). As a result, monovalent sugar ligands do not display high binding affinity for the ASGR and multiple interactions with the hetero-oligomeric receptor are required to enhance avidity. In some ways, interactions of 1 GalNAc ASOs can be rationalized using an avidity model where two weak ligands (GalNAc sugar and ASO) which bind at non-overlapping sites, are ligated to create a high-affinity ligand (38).

The generally accepted stoichiometry for ASGR1 and ASGR2 is 2:1. However, others have reported stoichiometries ranging from 2:1 to 5:1 for ASGR1 and ASGR2 based on immunoprecipitation assays (39). Thus, our binding data can be rationalized by models where the relatively long oligonucleotide spacer helps the GalNAc sugars bind multiple receptors on the cell surface or that the functional ASGR is a loosely held aggregate of 2–5 ASGR1 units. Previous work has also shown that cells over-expressing ASGR1 are capable of robust internalization of ASGR ligands into cells (40). ASGR1 possesses the carbohydrate binding pocket as well as the cytoplasmic signals to bind clathrin adaptor proteins (4). ASGR2 is not required for either function and possibly just serves to aggregate ASGR1 on the cell-surface which enhances avidity of the relatively weak monovalent binding interaction.

### ASGR knockout confirms proposed uptake mechanism

The contribution of ASGR towards uptake of PS ASOs was further confirmed in hepatocytes and in ASGR knockout mice where loss of ASGR expression coincided with loss of activity for both, GalNAc-conjugated and unconjugated ASOs. However, ASO activity was not completely ablated in ASGR knockout mice indicating that other pathways for ASO uptake are operative in these cells. Presumably, the ability of the PS backbone to enhance ASO protein binding promotes interactions with other cell-surface proteins which allows the ASO to be internalized into cells via multiple pathways.

## CONCLUSION

Conjugation with GalNAc sugars can improve the potency of second-generation ASOs in animals, as a direct result of enhanced delivery of the modified ASOs to hepatocytes via binding to ASGR. The optimal structure for efficient delivery is a single stranded ASO conjugated to two or three GalNAc sugars, as its potency is not affected by the composition of the ASO backbone. However, single stranded ASOs conjugated to a single GalNAc sugar also possess therapeutic potential for enhancing ASO potency via targeted delivery to the liver. The insights gained from this study support other efforts to further characterize properties of GalNAc-conjugated and unconjugated ASOs using the validated FP assay for high-throughput screening of potential therapeutics. GalNAc-conjugated ASOs represent an advanced therapeutic platform for liver-targeted delivery and are progressing into human clinical trials (1). Such enhancements in ASO potency and targeted delivery can significantly improve therapeutic index and reduce therapy costs for liver targeted ASO therapeutics. Our results also provide insights into how backbone charge and chemical composition assist in the binding and internalization of highly polar anionic single stranded oligonucleotides into cells and tissues.

## Supplementary Material

Supplementary Data
