# Supplementary material for: Characterizing the effect of GalNAc and phosphorothioate backbone on binding of antisense oligonucleotides to the asialoglycoprotein receptor
Source: Nucleic Acids Res. 2017 Feb 3;45(5):2294–306. doi: 10.1093/nar/gkx060 (PMC5389643; doi:10.1093/nar/gkx060)
Supplement: Supplementary Data [file gkx060_Supp.pdf]

Supporting Information

Characterizing the effect of GalNAc and phosphorothioate backbone on binding of antisense oligonucleotides to the asialoglycoprotein receptor

Karsten Schmidt, Thazha P. Prakash, Aaron J. Donner, Garth A. Kinberger, Hans J. Gaus, Audrey Low, Michael E. Østergaard, Melanie Bell, Eric E. Swayze, Punit P. Seth*

Ionis Pharmaceuticals Inc., 2855 Gazelle Court, Carlsbad, CA 92010

* To whom correspondence should be addressed:
Punit Seth
Tel: 760-603-2587
pseth@ionisph.com

Supplementary contents

Table S1. Chemical composition and analytical data for ASOs

Scheme S1. Synthesis of Tracer

Figure S1 – Additional details for FP assay

Figure S2 – Kinetics of apolipoprotein C-III (apoCIII) mRNA knockdown in hepatocytes

Figure S3 – SRB1 ASOs do not reduce GCGR mRNA in a non-specific manner in ASGR KO mice.

Figure S4 – Hypothetical model showing how a 1 GalNAc ASO conjugate can interact with the extracellular domain of ASGR.

Table S1. Chemical composition and analytical data for ASOs

ASO #	Target	Sequence	Calcd. Mass	Obsvd. Mass	%UV Purity
1	Mouse SRB1	GN3-AGCTTCAGTCATGACTTCCTT	8978.1	8977.3	99
2	Mouse SRB1	GN2-AGCTTCAGTCATGACTTCCTT	8532.6	8531.6	98
3	Mouse SRB1	GN1-AGCTTCAGTCATGACTTCCTT	8257.3	8256.3	98
4	Mouse SRB1	GCTTCAGTCATGACTTCCTT	7164.2	7164	93
5	Mouse SRB1	GN3-AGCTTCAGTCATGACTTCCTT	8881.7	8881	98
6	Mouse SRB1	GN2-AGCTTCAGTCATGACTTCCTT	8436.2	8435.3	97
7	Mouse SRB1	GN1-AGCTTCAGTCATGACTTCCTT	8160.9	8160.1	98
8	Mouse SRB1	GCTTCAGTCATGACTTCCTT	7247	7246.7	93
9	Mouse SRB1	GN3-AGCTTCAGTCATGACTTCCTT	8407.8	8406.6	97
10	Mouse SRB1	GN2-AGCTTCAGTCATGACTTCCTT	7962.3	7961.1	98
11	Mouse SRB1	GN1-AGCTTCAGTCATGACTTCCTT	7687	7685.9	98
12	Mouse SRB1	GCTTCAGTCATGACTTCCTT	7086.3	7085.6	89
13	Mouse SRB1	GN3-AGCTTCAGTCATGACTTCCTT	8672.8	8671.4	97
14	Mouse SRB1	GN2-AGCTTCAGTCATGACTTCCTT	8227.3	8226	97
15	Mouse SRB1	GN1-AGCTTCAGTCATGACTTCCTT	7952	7950.7	97
16	Mouse SRB1	GCTTCAGTCATGACTTCCTT	6859	6857.8	96
17	Mouse SRB1	GN3-GCTTCAGTCATGACTTCCTT	8383.7	8382.4	87
18	Mouse SRB1	GN2-GCTTCAGTCATGACTTCCTT	7938.2	7937.3	89
19	Mouse SRB1	GN1-GCTTCAGTCATGACTTCCTT	7662.9	7661.6	88
20	none	GN3-TT	2066.1	2065.3	94
21	none	GN3-TTTTTT	3043	3042.4	98
22	none	GN3-TTTTTTTTTT	4644.3	4643.6	97
23	none	GN3-TTTTTTTTTTTTTTTTTTTT	7846.9	7846.2	98
24	none	GN1-AA	1160.1	1159.8	98
25	none	GN1-AAAAA	2164	2163.4	93
26	none	GN1-AAAAAAAAAA	3810.4	7060.2	95
27	none	GN1-AAAAAAAAAAAAAAAAAAAA	7103.2	7101.9	93
28	Mouse SRB1	GCTTCAGTCATGACTTCCTT-GN-GN-GN	8966	8966.1	95
29	Mouse SRB1	GN-GCTTCAGTCATGACTTCCTT	7660.7	7659.1	93
30	Mouse SRB1	GCTTCAGTCATGACTTCCTT-GN	7660.7	7659.9	98
31	Mouse SRB1	GCTTCAGTCATGACTTCCTT-GN-GN	8157.1	8156.5	88
32	Mouse SRB1	GN-GCTTCAGTCATGACTTCCTT-GN	8157.1	8156.2	94
33	none	CGAAGTCAGTACTGAAGGAA	6601.8	6601.1	88
34	none	CGAAGTCAGTACTGAAGGAA-GN	7098.2	7097.6	84
35	none	GN-CGAAGTCAGTACTGAAGGAA	7098.2	7097.6	84
36	none	GN-CGAAGTCAGTACTGAAGGAA-GN	7594.7	7594	84
37	none	CGAAG*UCAGUACUGA*AGGAA	6745.9	6744.6	98
38	Mouse GCGR	GN3-ATGGCTTCTCCATCATATCC	8666.9	8666.2	95

39	Mouse GCGR	ATGGCTTCTCCATCATATCC	7147.2	7146.6	95
40	Mouse apoCIII	CAGCTTTATTAGGGACAGCA	7243.3	7242.4	95
41	Mouse apoCIII	GN3- CAGCTTTATTAGGGACAGCA	8837.7	8837.2	98

GNX = 0, 1, 2 or 3 GalNAc; orange = 2'-O-methoxyethyl (MOE) nucleosides; red = 2'-O-methyl; black = DNA; italicized letters RNA; all phosphorothioate (PS) backbone, underlined letters = phosphodiester (PO) linkage; green = phosphorodiamidate linked morpholinos. All GalNAc conjugates were attached to the ASO using a phosphodiester linkage.

Scheme S1. Synthesis of Tracer

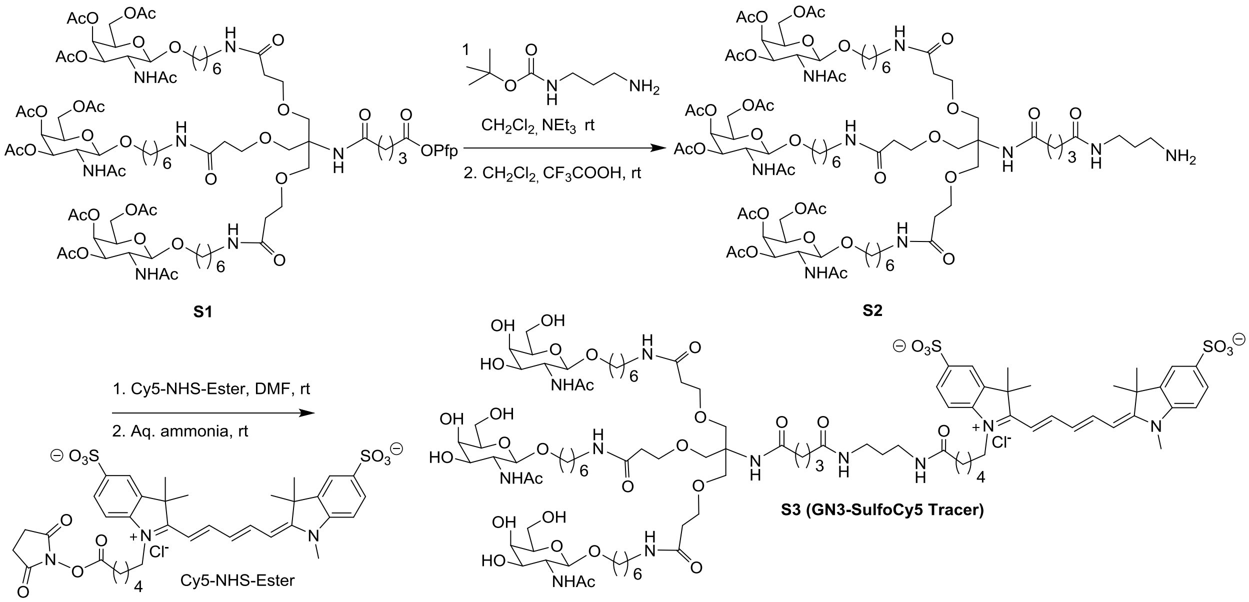

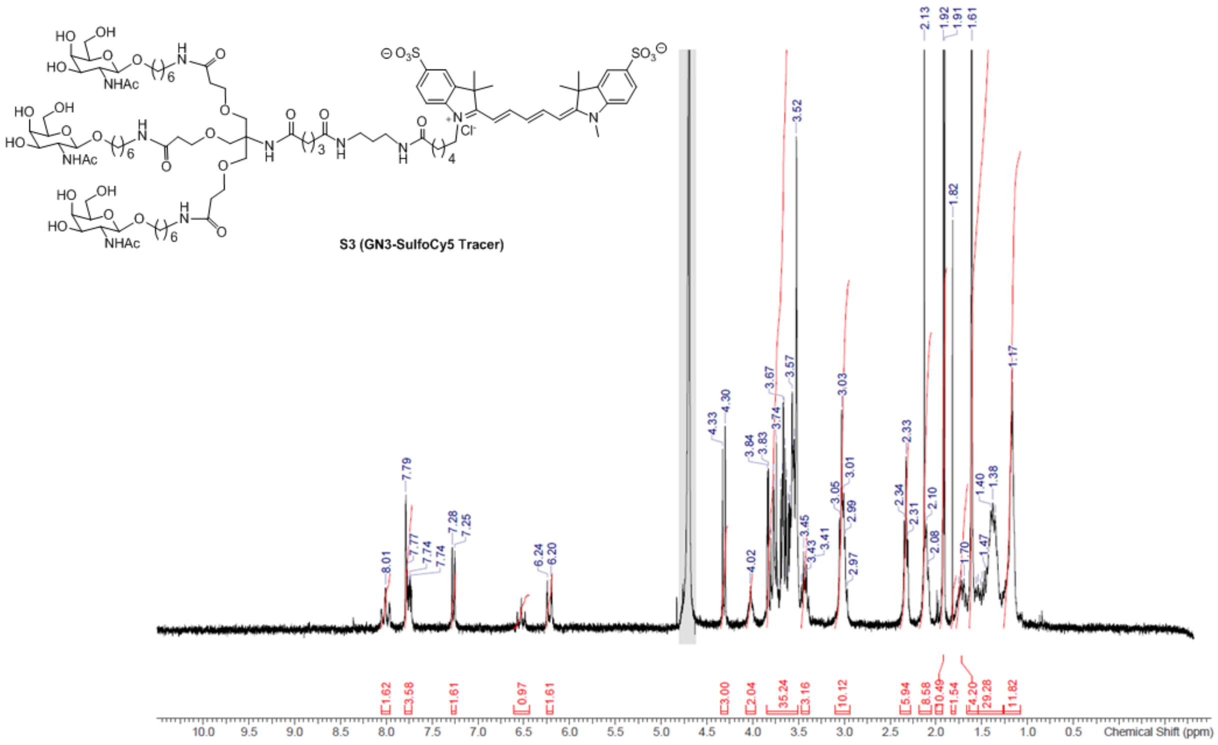

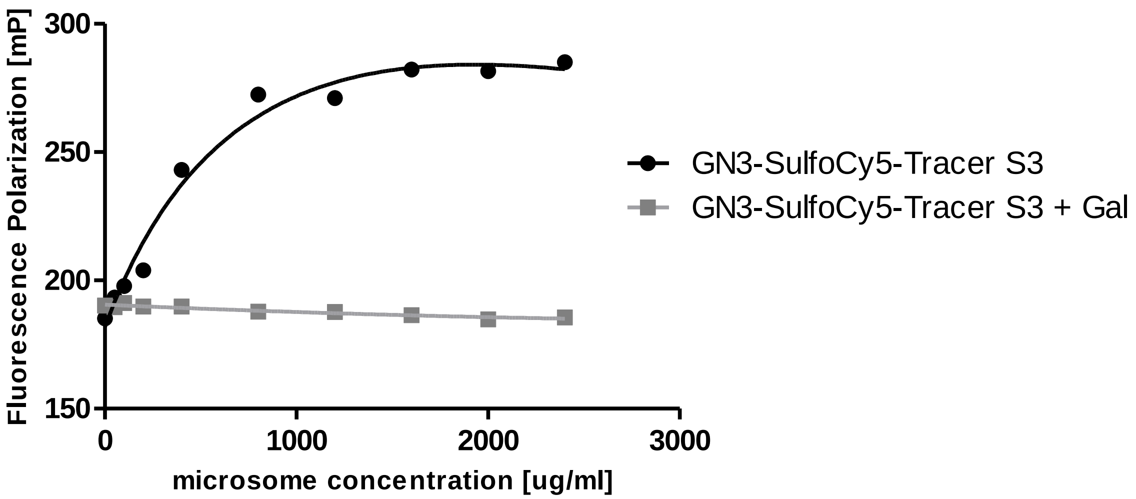

Figure S1. Binding of 1.25 nM GN3-sulfoCy5 tracer S3 to ASGR in microsome preparation. Addition of 1 mM galactose was able to abolish binding curve demonstrating specific binding of GN3-SulfoCy5-Tracer S3.

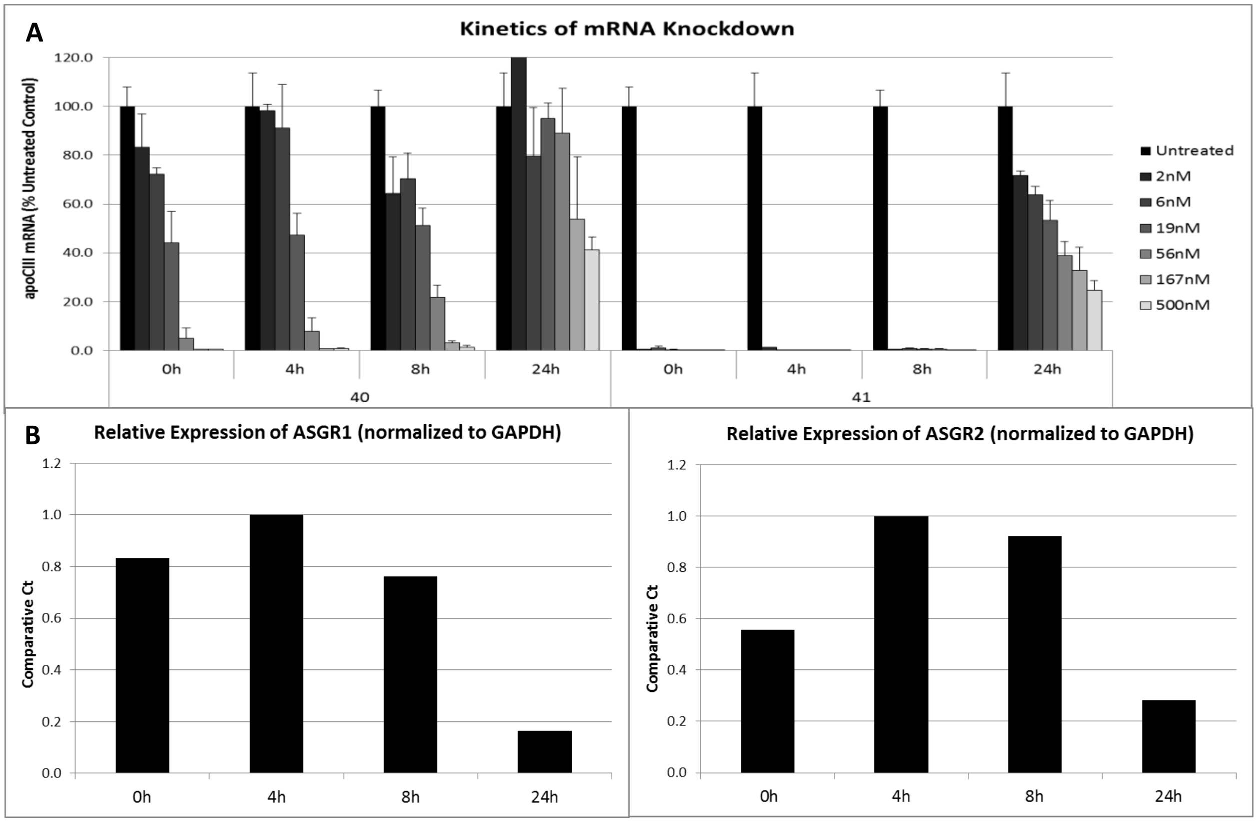

Figure S2. (A) Kinetics of apolipoprotein C-III (apoCIII) mRNA knockdown in hepatocytes. Hepatocytes were plated and treated with ASOs 4, 8 and 24 hours after plating. The cells were incubated for an additional 16 hours post oligo addition after which RNA was isolated and knockdown of apoCIII mRNA was measured using qRT-PCR. apoCIII ASOs **40** & **41**: CAGCTTTATTAGGGACAGCA; GN3-CAGCTTTATTAGGGACAGCA; GN3 = GalNAc3; orange = 2'-O-methoxyethyl (MOE) nucleosides; all phosphorothioate (PS) backbone. qRT-PCR was performed using Agpath ID One-Step qRT-PCR kit (Life Technologies) on the StepOne 96-well Real Time PCR system (Applied Biosystems) using an apoCIII specific Taqman primer probe set primers: 5'-TGCAGGGCTACATGGAACAA-3' and 5'- CGGACTCCTGCACGCTACTT-3'. Probe with 5' fluorescein and 3' TAMRA: 5'-CTCCAAGACGGTCCAGGATGCGC-3'.

(B) ASGR1 and ASGR2 expression normalized to GAPDH showed reduced mRNA expression after 24 hours of plating in culture. qRT-PCR was performed Agpath ID One-Step qRT-PCR kit (Life Technologies) on the StepOne Plus 96-well Real Time PCR system (Applied Biosystems). ASGR1, ASGR2 and GAPDH were measured using gene specific Taqman primer probe sets. ASGR1 primers:5'-GCCATCATGACAAAGGATTATCAA-3' and 5'-CGGCCCTCTCCGGAGTT-3'. Probe: with 5' fluorescein and 3' TAMRA: 5' CCAGCACCTGGACAATGATAATGACCATC-3'. ASGR2 primers: 5'-ACCTGTCAGCTGGCGTACTTC-3' and 5'-CCCGAACTCCACCCAGTTAAC-3'. Probe: with 5' fluorescein and 3' TAMRA: 5' CAATGGCACGGAATGCTGCCC-3'. GAPDH primers: 5'- GGCAAATTCAACGGCACAGT-3' and 5'- GGGTCTCGCTCCTGGAAGAT-3'. Probe: with 5' fluorescein and 3' TAMRA: 5'-AAGGCCGAGAATGGGAAGCTTGTCATC-3'

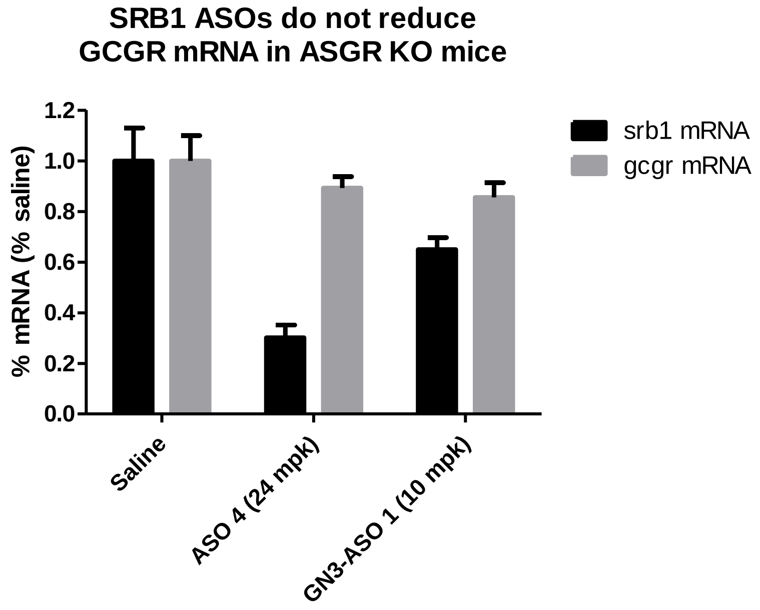

Figure S3 – SRB1 ASOs do not reduce GCGR mRNA in a non-specific manner in ASGR KO mice. ASGR KO mice (n=3/group) were injected subcutaneously with GN3-ASO 1 (10 mg/kg) or unconjugated ASO 4 (24 mg/kg) targeting SRB1 mRNA. Mice were sacrificed after 72 hours and livers were homogenized and reductions of SRB1 and GCGR mRNA were quantified by qRT-PCR.

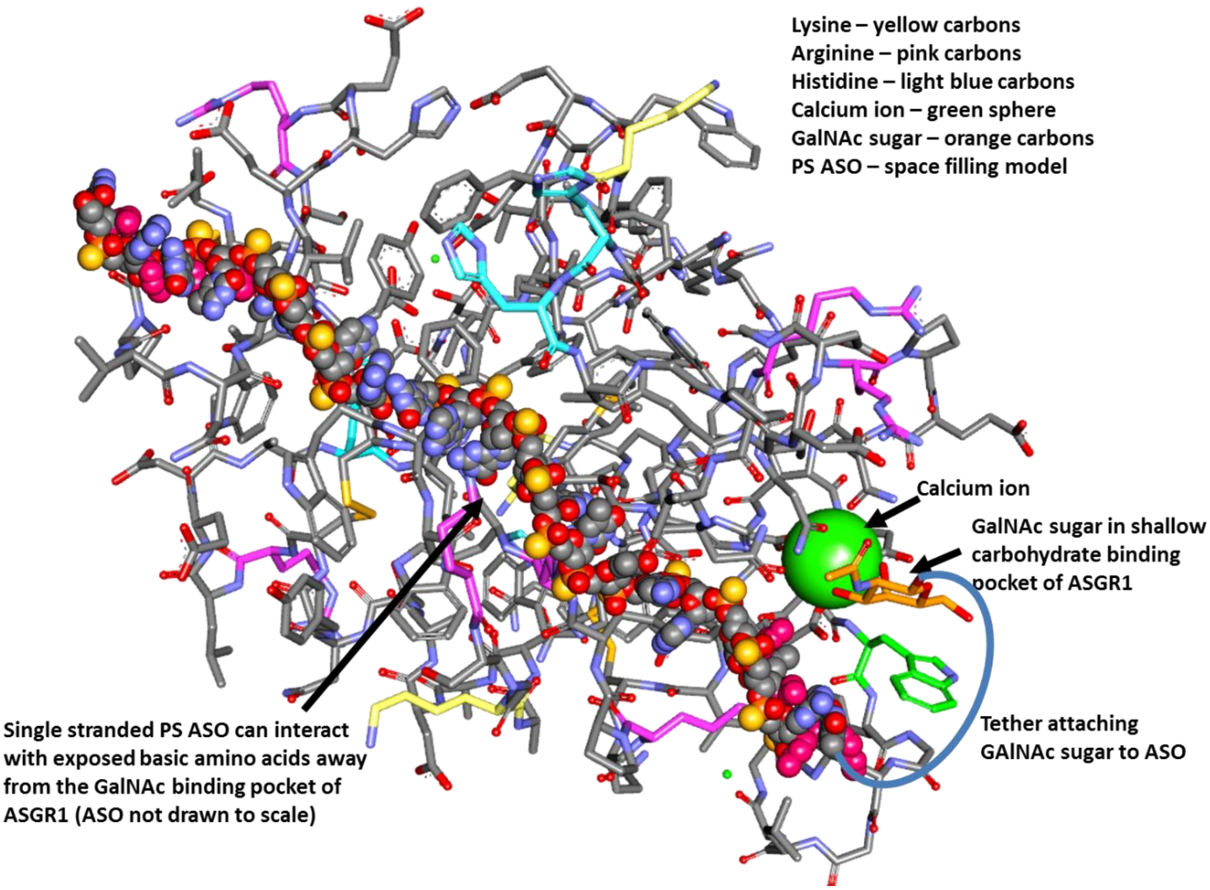

Figure S4 – Hypothetical model showing how a 1 GalNAc ASO conjugate can interact with the extracellular domain of ASGR1 (Meier et al, Crystal structure of the carbohydrate recognition domain of the H1 subunit of the asialoglycoprotein receptor. *J.Mol. Biol.,* 2000, **300**, 857-865). ASGR is a constitutively internalizing receptor which localizes in clathrin coated pits on the basolateral membrane of hepatocytes. The GalNAc sugar binds a calcium ion within the shallow solvent exposed sugar binding pocket of ASGR1 while the single stranded ASO can interact with several solvent exposed basic amino acids side chains of ASGR1. In addition, the nucleobases can potentially participate in stacking hydrophobic interactions with aromatic amino acid side-chains. These interactions can presumably be facilitated by the flexible single stranded and negatively charged ASO. Association of GalNAc conjugated and unconjugated ASOs with the ASGR can further promote internalization into hepatocytes via clathrin mediated endocytosis.
